# Identification of functional gene modules by integrating multi-omics data and known molecular interactions

**DOI:** 10.3389/fgene.2023.1082032

**Published:** 2023-01-24

**Authors:** Xiaoqing Chen, Mingfei Han, Yingxing Li, Xiao Li, Jiaqi Zhang, Yunping Zhu

**Affiliations:** ^1^ Basic Medical School, Anhui Medical University, Hefei, China; ^2^ National Center for Protein Sciences (Beijing), Beijing Proteome Research Center, Beijing Institute of Lifeomics, Beijing, China; ^3^ Central Research Laboratory, Peking Union Medical College Hospital, Chinese Academy of Medical Sciences and Peking Union Medical College, Beijing, China

**Keywords:** multi-omics integration, gene module detection, proteomic, transcriptomic, genomic

## Abstract

Multi-omics data integration has emerged as a promising approach to identify patient subgroups. However, in terms of grouping genes (or gene products) into co-expression modules, data integration methods suffer from two main drawbacks. First, most existing methods only consider genes or samples measured in all different datasets. Second, known molecular interactions (e.g., transcriptional regulatory interactions, protein–protein interactions and biological pathways) cannot be utilized to assist in module detection. Herein, we present a novel data integration framework, Correlation-based Local Approximation of Membership (CLAM), which provides two methodological innovations to address these limitations: 1) constructing a trans-omics neighborhood matrix by integrating multi-omics datasets and known molecular interactions, and 2) using a local approximation procedure to define gene modules from the matrix. Applying Correlation-based Local Approximation of Membership to human colorectal cancer (CRC) and mouse B-cell differentiation multi-omics data obtained from The Cancer Genome Atlas (TCGA), Clinical Proteomics Tumor Analysis Consortium (CPTAC), Gene Expression Omnibus (GEO) and ProteomeXchange database, we demonstrated its superior ability to recover biologically relevant modules and gene ontology (GO) terms. Further investigation of the colorectal cancer modules revealed numerous transcription factors and KEGG pathways that played crucial roles in colorectal cancer progression. Module-based survival analysis constructed four survival-related networks in which pairwise gene correlations were significantly correlated with colorectal cancer patient survival. Overall, the series of evaluations demonstrated the great potential of Correlation-based Local Approximation of Membership for identifying modular biomarkers for complex diseases. We implemented Correlation-based Local Approximation of Membership as a user-friendly application available at https://github.com/free1234hm/CLAM.

## 1 Introduction

Increasing attention has been devoted to the integration of multi-omics data to discover coherent biological signatures. In a comprehensive review of multi-omics data integration methods, Huang et al. (Huang et al., 2017) categorized the existing algorithms into four classes: matrix factorization methods (e.g., NMF (Zhang et al., 2011; Zhang et al., 2012) and iCluster (Shen et al., 2012), Bayesian methods (e.g., MDI (Kirk et al., 2012), BCC (Lock and Dunson, 2013) and CONEXIC (Akavia et al., 2010), network-based methods (e.g., SNF (Wang et al., 2014), MoGCN (Li et al., 2022) and Lemon-tree (Bonnet et al., 2015), and multi-step analysis (e.g., CNAmet (Louhimo and Hautaniemi, 2011) and iPAC (Aure et al., 2013). These methods can discover patient subgroups when using samples as clustering objects and genes (or gene products) as clustering features, or identify co-expressed gene modules by exchanging the clustering objects and features.

However, most of the existing methods are particularly suitable for patient subtyping. Although some methods can be applied to gene module detection, such as jNMF (Zhang et al., 2012), iNMF (Yang and Michailidis, 2016), moCluster (Meng et al., 2016), iCluster, CONEXIC, Lemon-tree (Bonnet et al., 2015), etc., they suffer from two main drawbacks. First, most methods are limited in terms of input data, requiring the datasets from different sources to share the same clustering objects (genes) or features (samples). For example, jNMF and iNMF require the input data to share the same samples, iCluster and moCluster require the input data to share the same genes. Second, because co-expressed genes are often functionally related or co-regulated, known molecular interactions (e.g., transcriptional regulatory interactions, protein–protein interactions and biological pathways) are valuable for improving module detection. Although there are approaches that integrate multi-omics data and molecular interactions, most of these methods are aimed at biomarker discovery. For example, EMOG (Schulte-Sasse et al., 2021) integrates multi-omics data and protein–protein interaction networks to identify new cancer genes. ModulOmics (Silverbush et al., 2019) integrates multi-omics data and molecular networks to improve the identification of cancer driver modules. To our knowledge, molecular interactions are rarely used to improve the identification of co-expressed gene modules.

Herein, we present a novel analytical framework referred to as Correlation-based Local Approximation of Membership (CLAM), which employs three methodological innovations to address the above challenges. First, CLAM constructs a k-nearest neighbor (KNN) matrix for each dataset and combines them into a trans-omics neighborhood matrix. The combined matrix includes all genes measured in at least one dataset. Therefore, this step does not require different datasets to share the same genes or samples. Second, CLAM uses various known molecular interactions, such as transcriptional regulatory interactions, protein–protein interactions and biological pathways, to adjust the neighborhood matrix. Third, CLAM applies a local approximation procedure to define gene modules and performs module-based survival analysis to evaluate module–disease relationships. We have implemented CLAM as a user-friendly application with extensive interactive interfaces available at https://github.com/free1234hm/CLAM.

By applying CLAM and state-of-the-art module detection methods to human colorectal cancer (CRC) and mouse B-cell differentiation multi-omics data obtained from The Cancer Genome Atlas (TCGA), Clinical Proteomics Tumor Analysis Consortium (CPTAC), Gene Expression Omnibus (GEO) and ProteomeXchange database, we demonstrated that CLAM showed the highest precision, recall, relevance and recovery metrics in recovering biologically relevant modules and identified the highest number of gene ontology (GO) terms in enrichment analysis. Additionally, further investigation of the CRC modules revealed numerous transcription factors (TFs) and KEGG pathways that played crucial roles in CRC progression. Module-based survival analysis constructed four gene networks significantly correlated with CRC survival. In contrast to traditional survival genes, which affect patient survival based on their own expression levels, genes in the four survival-related networks affect patient survival based on the levels of their co-expression. We found that many genes in these networks played crucial roles in cancer progression and could serve as potential prognostic biomarkers. Overall, our results demonstrated the superior ability of CLAM in reconstructing modular structure from multi-omics data and identifying modular biomarkers for CRC.

## 2 Materials and methods

**FIGURE 1:**
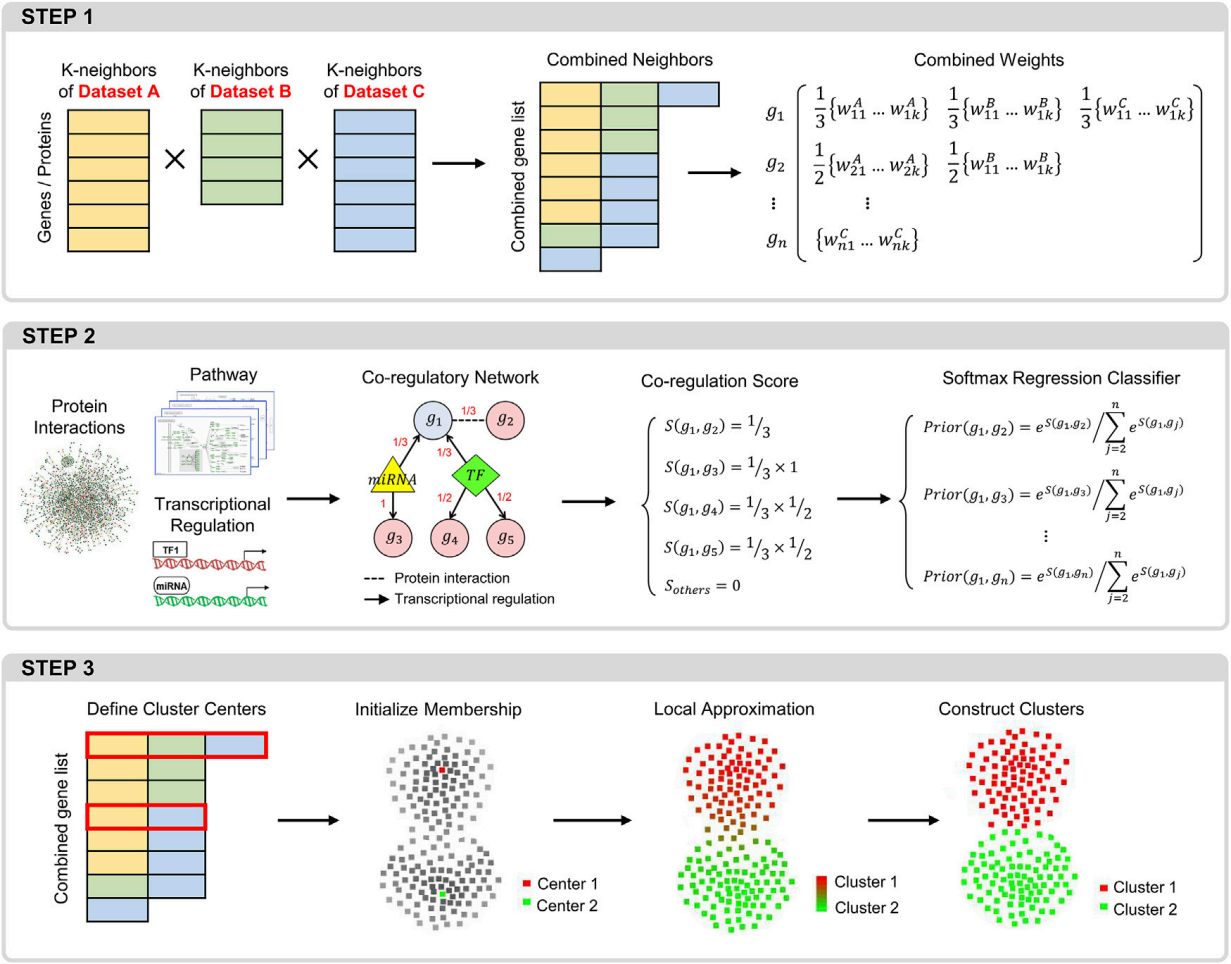
Overview of the CLAM workflow. Step 1: procedure for integrating the KNN matrices of different datasets into a global neighborhood matrix. Step 2: procedure for calculating the prior correlation probabilities between each gene and its neighbors in the neighborhood matrix. Step 3: the local approximation procedure for identifying gene modules.

CLAM includes modules with the following three key functions (Figure 1): 1) constructing a trans-omics neighborhood matrix by integrating the k-nearest neighbor matrices obtained from different data sources; 2) using known molecular interactions to adjust the combined neighborhood matrix; and 3) using a local approximation procedure to define gene modules.

### 2.1 Construction of the trans-omics neighborhood matrix

In each dataset, we first calculate the similarity between each pair of objects (genes or proteins) and extract the k (10 as default) nearest neighbors for each object. The similarity measure can be Euclidean distance, mutual information and Pearson correlation coefficient, etc. Second, the similarity measures between each object and its nearest neighbors are used to calculate a set of weights $W=\left\{w_{1},\ldots ,w_{k}\right\}$ . The weight between genes x and y is calculated as $w_{xy}=S_{xy}/\underset{z\in KNN\left(x\right)}{\sum }S_{xz}$ , where $S_{xy}$ represents the similarity measures between genes x and y . The neighbors that have higher similarities are given higher weights and $\underset{y\in KNN\left(x\right)}{\sum }w_{xy}=1$ . Third, we combine the KNN matrices of different datasets into a global neighborhood matrix, which includes the neighborhood information for all the genes measured in at least one dataset. If a gene has measurements in m datasets, it has m×k neighbors in the combined matrix with the previous weights divided by m . Therefore, different genes may have different numbers of neighbors (Figure 1 Step 1). Finally, the duplicated neighbors and their weights are merged. This step ensures that the duplicated neighbors are given higher weights.

### 2.2 Calculation of the prior correlation probability

For each gene in the combined neighborhood matrix ( $g_{1}$ in Figure 1 Step 2), we construct a co-regulatory network using protein–protein interactions (PPIs), transcriptional regulatory interactions (*via* TFs or miRNAs) and KEGG pathways. This network consists of $g_{1}$ and its neighbors which directly interact with $g_{1}$ or share the same transcriptional regulators with $g_{1}$ . Second, we calculate a co-regulation score between $g_{1}$ and each neighbor according to the network structure. Assuming that $g_{1}$ binds to $n_{1}$ miRNAs; $n_{2}$ TFs and directly interacts with $n_{3}$ genes through PPIs, the edges directly connected to $g_{1}$ have the same weight $1/\left(n_{1}+n_{2}+n_{3}\right)$ . Given a miRNA (or TF) that binds to $n_{4}$ neighbors of $g_{1}$ , the weight between the miRNA and each target $g_{i\in \left[1,n_{4}\right]}$ is $1/n_{4}$ . Finally, the co-regulation score between $g_{1}$ and $g_{i}$ is $1/\left(n_{1}+n_{2}+n_{3}\right)\times 1/n_{4}$ . Neighbors not included in the co-regulatory network are scored zero. Third, we calculate the prior probability between $g_{1}$ and each neighbor using softmax regression (a generalization of logistic regression that we can use for multi-class classification). Finally, the weights between gene x and its neighbors are transformed to $\left(w_{xy}\times {prior}_{xy}\right)$ , where $y\in KNN\left(x\right)$ . This step assures that functionally related or co-regulated genes are given relatively high weights.

### 2.3 Identification of gene modules

We followed the local approximation process proposed by Fu et al. (Fu and Medico, 2007) to define gene modules. First, the density of one object is calculated as the average similarity measure between this object and its k -nearest neighbors. Second, the densities of all objects are used to identify cluster centers and outliers: 1) one object is defined as a cluster center when its density is higher than that of all objects in its neighborhood and 2) one object is defined as an outlier when its density is lower than that of all objects in its neighborhood. The higher k is, the fewer cluster centers will be identified; as a consequence, fewer clusters will be generated. Third, we define a membership vector for each object. Assuming that we have identified M cluster centers, the membership vector of each object x is represented as $p\left(x\right)=\left\{p_{1}\left(x\right),\ldots ,p_{M+1}\left(x\right)\right\}$ , in which each element $p_{i}\left(x\right)$ indicates the membership degree of x to cluster i and the last element indicates the probability that x is an outlier.

Next, we initialize the membership vector of each object. First, each cluster center is assigned a unique membership vector, where only the element corresponding to its own cluster is 1 and the other elements are 0. Second, all outliers are assigned the same membership vector, in which the last element is 1 and the other elements are 0. Third, for all other objects, the elements in each vector are set to the same value $1/\left(M+1\right)$ . Subsequently, through an iteration process, we update the membership vector of each object (except for cluster centers and outliers) using its linear approximation, which is calculated by combining its nearest neighbors’ membership vectors, namely, $p\left(x\right)\approx \underset{y\in KNN\left(x\right)}{\sum }w_{xy}p\left(y\right)$ , where w is the weight matrix produced by integrating multi-omics data and known relationships. The iteration process is terminated when the overall difference between all membership vectors and their approximations is minimized, which is calculated as follows:

$$E=\underset{x\in X}{\sum }\|p\left(x\right)-\underset{y\in KNN\left(x\right)}{\sum }w_{xy}p\left(y\right){\|}^{2}$$

where each term is the difference between the membership vector $p\left(x\right)$ and the linear approximation of $p\left(x\right)$ by its neighbors $\underset{y\in KNN\left(x\right)}{\sum }w_{xy}p\left(y\right)$ . Finally, each object is assigned to the cluster with the highest score in the final membership vector.

### 2.4 Data collection and preprocessing

Mouse and human multi-omics data were used for the evaluation study. The mouse datasets include RNA-seq data from GEO (GSE75417) and MS data from PRIDE (PXD003263). Both datasets share the same samples collected at six time points during the differentiation process of mouse pre-B-cells. The human data include RNA-seq and MS data of CRC patients obtained from TCGA and CPTAC. The RNA-seq and MS data include 497 and 90 samples, respectively, in which 47 samples are collected from the same patients. Because some existing methods (e.g., iNMF and jNMF) require the input data to share the same set of samples, only the 47 samples from the same patients were used in the evaluation study. Among the initial genes or proteins measured in different omics datasets, we removed those with more than 20% missing values. The remaining missing values were filled using the KNN imputation method. The processed expression matrices are included in the CLAM toolkit as test data.

### 2.5 Evaluation metrics

We followed the evaluation pipeline proposed by Saelens et al. (Saelens et al., 2018) to compare the performance of CLAM and existing methods. First, we collected various types of known modules, including 1599 human and 1078 mouse miRNA modules extracted from known miRNA–target interactions (Chen and Wang, 2020), 795 human and 1349 mouse TF modules extracted from known TF–target interactions (Cahan et al., 2014; Han et al., 2018; Zhang et al., 2020), and 335 pathway modules from the KEGG database (Kanehisa et al., 2017). Second, we calculated the recovery, relevance, recall, and precision metrics by comparing the known modules with a set of detected modules. These scores have been previously used in several evaluation studies (Prelic et al., 2006; Amigo et al., 2009; Eren et al., 2013; Saelens et al., 2018). If G represents all genes, M a set of known modules, $M^{\prime }$ a set of observed modules, $M\left(g\right)$ the modules that contain gene g , and $E\left(g,M\right)$ the set of genes that are included with g in at least one module of M (including g itself), the precision is defined as follows:

$$Precision=\frac{1}{\left|G\right|}\underset{g\in G}{\sum }\left[\frac{1}{\left|E\left(g,M^{\prime }\right)\right|}\underset{g^{\prime }\in E\left(g,M^{\prime }\right)}{\sum }\frac{\min\left(\left|M^{\prime }\left(g\right)\cap M^{\prime }\left(g^{\prime }\right)\right|,\left|M\left(g\right)\cap M\left(g^{\prime }\right)\right|\right)\times \Phi \left(g,g^{\prime }\right)}{\left|M^{\prime }\left(g\right)\cap M^{\prime }\left(g^{\prime }\right)\right|}\right]$$

where *Φ* $\left(g,g^{\prime }\right)=\frac{1}{\left|M^{\prime }\left(g,g^{\prime }\right)\right|}\underset{m^{\prime }\in M^{\prime }\left(g,g^{\prime }\right)}{\sum }\max_{m\in M\left(g,g^{\prime }\right)}Jaccard\left(m^{\prime },m\right)$ . Recall is calculated in the same way but with $M^{\prime }$ and M switched. In addition, relevance is defined as ***Relevance*** $=\frac{1}{\left|M^{\prime }\right|}\underset{m^{\prime }\in M^{\prime }}{\sum }\max_{m\in M}Jaccard\left(m^{\prime },m\right)$ , and recovery is calculated in the same way but with $M^{\prime }$ and M switched. Third, before combining the four scores, we normalized every score by dividing it by an average score of 500 permuted versions of the known modules. Finally, we calculated the harmonic mean between the normalized versions of all four scores to obtain an overall score.

### 2.6 Module-based survival analysis

In traditional survival analysis, patients are ranked according to the expression of a specific gene. The log-rank test is then applied to determine whether there is a significant survival difference between the top and bottom half (or 1/4) of the ranked patients. However, the differential expression of a single gene is not the only factor that affects patient survival, and the traditional approach ignores the potential effects of differential regulation between multiple genes. In this study, we present a module-based survival analysis approach to identify the sets of genes whose co-expression levels are significantly correlated with patient survival.

Given a gene module of $M=\left(G,S,v\right)$ , where $G=\left\{g_{1},g_{2},g_{3}\right\}$ represents the genes included in M , $S=\left\{s_{1},\ldots ,s_{N}\right\}$ represents all patient samples, and v:3×N represents the expression-value matrix. Assuming that the expression profile of $g_{1}$ ( $\left\{v_{11},\ldots ,v_{1N}\right\}$ ) is positively correlated with that of $g_{2}$ ( $\left\{v_{21},\ldots ,v_{2N}\right\}$ ) and negatively correlated with that of $g_{3}$ ( $\left\{v_{31},\ldots ,v_{3N}\right\}$ ). First, z-score normalization is applied to $\left\{v_{11},\ldots ,v_{1N}\right\}$ , $\left\{v_{21},\ldots ,v_{2N}\right\}$ , and $\left\{v_{31},\ldots ,v_{3N}\right\}$ , which ensures the same weight of the three genes. Second, the normalized expression of $g_{3}$ is transformed to $\left\{-v_{31},\ldots ,-v_{3N}\right\}$ , which ensures that the three genes show theoretically similar expression patterns. Third, for each patient, $s_{i}$ ( $i\in \left[1,N\right]$ ), we calculate the standard deviation ( $\sigma_{i}$ ) of the transformed expression values of the three genes ( $v_{1i}$ ; $v_{2i}$ ,; $-v_{3i}$ ). The standard deviation $\sigma_{i}$ can represent the co-expression level of the three genes in patient $s_{i}$ . Genes are highly co-expressed in patients with low standard deviations and present lower co-expression in patients with high standard deviations. Finally, the log-rank test is applied to compare the survival curves between patients whose standard deviations were greater than the median *versus* those whose standard deviations were less than or equal to the median. A significant *p*-value indicates that the three genes affect patient survival based on their pairwise expression similarities.

## 3 Results

### 3.1 Method evaluation

**FIGURE 2:**
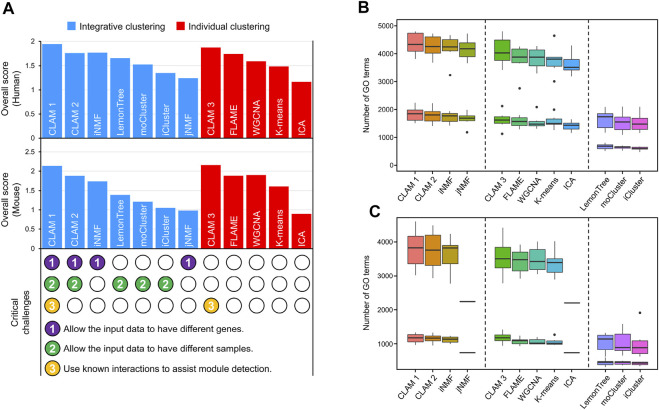
Comparison of 12 module detection methods, including 7 integrative clustering methods and 5 individual clustering methods. **(A)** The histograms show the agreement between the observed and known modules. Scores in the upper and middle histograms are generated by applying the tested methods to human and mouse data, respectively. The bottom shows whether the tested methods address three critical challenges of integrative clustering. **(B–C)** The number of significantly enriched GO terms in **(B)** human and **(C)** mouse modules with *p*-value thresholds of 0.01 and 0.05.

Using RNA-seq and MS data of human CRC and mouse B-cell differentiation (see ‘Data collection and preprocessing’), we conducted a comprehensive evaluation of 12 module detection methods, including 7 integrative clustering methods and 5 individual clustering methods. The integrative clustering methods included CLAM1 (using known molecular interactions to assist module detection), CLAM2 (without using known interactions), jNMF (Zhang et al., 2012), iNMF (Yang and Michailidis, 2016), Lemon-tree (Bonnet et al., 2015), moCluster (Meng et al., 2016) and iCluster (Shen et al., 2009). And the individual clustering methods included CLAM3 (applying the CLAM algorithm to individual datasets), independent component analysis (ICA) (Im et al., 2022), FLAME (Fu and Medico, 2007), K-means (Lin et al., 2004) and WGCNA (Langfelder and Horvath, 2008). Among these methods, CLAM1 is the only one that addresses three critical challenges, including allowing different samples in different datasets, allowing different genes in different datasets, and utilizing known molecular interactions to assist module detection (see the bottom of Figure 2A).

According to the evaluation pipeline described in MATERIALS AND METHODS, we scored the different methods by comparing their observed modules with sets of known modules obtained from TF/miRNA–target interaction networks and KEGG pathway database. Notably, because CLAM1 and CLAM3 use known interactions to assist model training, we used 5-fold cross-validation to differentiate the training and validation sets. First, the known modules were randomly divided into five parts. Second, CLAM1 and CLAM3 used four of those parts for training and reserved one-fifth for evaluation, while the other methods used each of the five folds to evaluate their performance. Finally, step 2 was repeated five times to calculate the average evaluation score for each method. The evaluation results revealed several interesting conclusions. First, CLAM1 and CLAM3 achieved the highest overall scores among all tested methods (Figure 2A), indicating that the utilization of known interactions significantly improved the consistency between the observed and known modules (although the interactions used were not included in the known modules). Second, CLAM2 outperformed most of the existing integrative clustering methods, but showed no significant advantage over FLAME or WGCNA. This indicated that the integration of datasets from different sources contributed little to the overall score. However, using another evaluation method, gene ontology (GO) enrichment analysis, we reached a different conclusion.

According to the number of significantly enriched GO terms, the tested methods could be divided into three categories (Figures 2B, C): methods that allow the input data to have different genes (CLAM1, CLAM2, iNMF and jNMF) performed best; methods that process each input dataset separately (CLAM3, FLAME, WGCNA and k-means) took second place; and methods that cluster the overlapping genes in the input data (Lemon-tree, moCluster and iCluster) identified the fewest GO terms. These results suggest that the number of significantly enriched GO terms is positively correlated with the total number of genes in the final modules. Therefore, the integrative clustering methods that allow the input data to have different genes are well suited for GO enrichment analysis because they cluster the union of genes in the input data. Additionally, we found a significant reduction in the number of GO terms identified by jNMF and ICA from the mouse data (Figure 2C). This is because both jNMF and ICA have a limitation that the number of modules must be less than the number of samples. However, there are only 18 samples in the mouse RNA-seq and MS data, which results in jNMF and ICA generating far fewer modules than the other methods.

In summary, the utilization of known molecular interactions can improve the agreement with known modules, while data integration can improve the discovery of functional annotations. This is why CLAM1, which integrates multi-omics data and known molecular interactions, performs best on both evaluation metrics.

### 3.2 Investigation of the resulting modules

**FIGURE 3:**
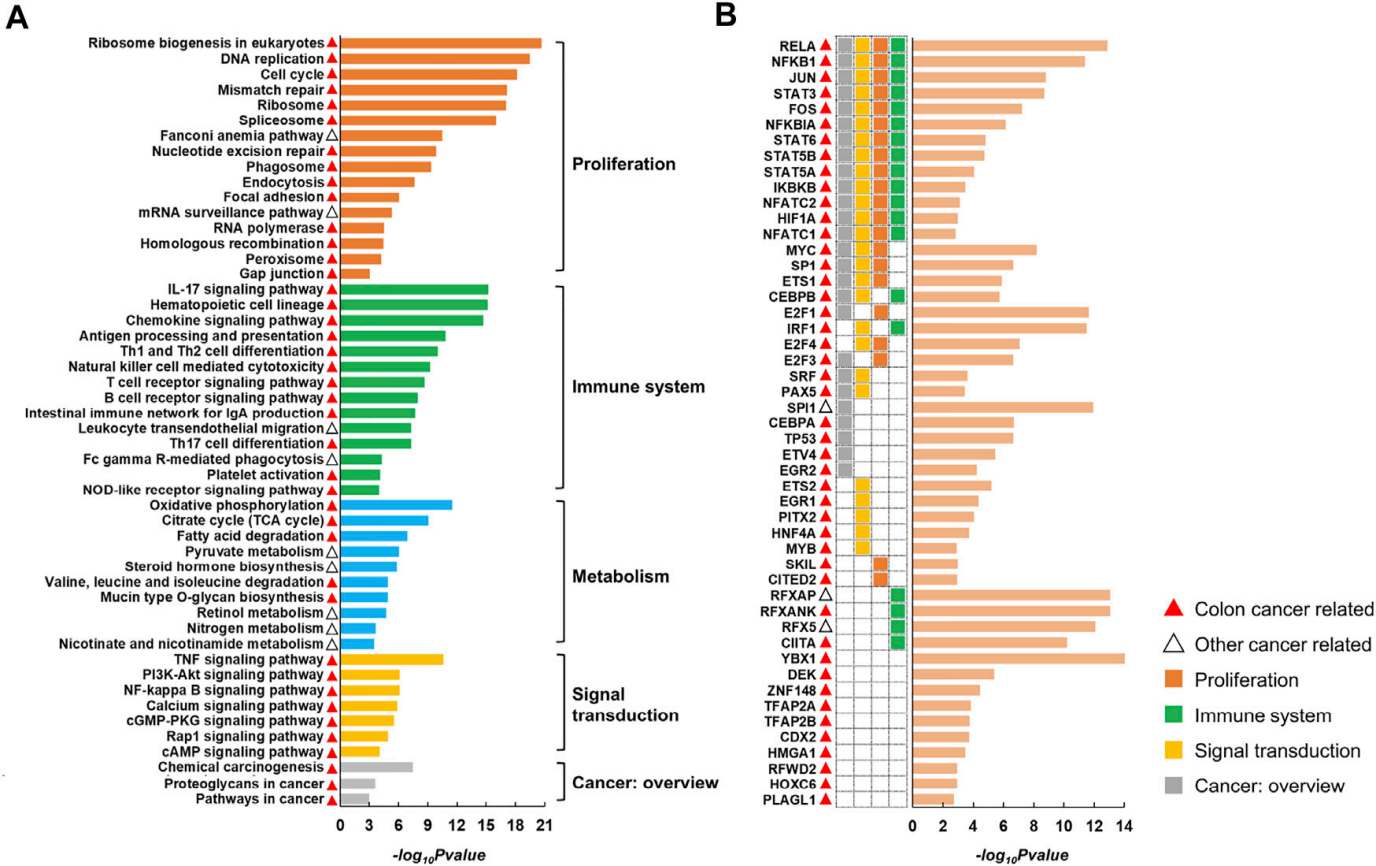
Representative pathways **(A)** and TFs **(B)** identified by enrichment analysis of CRC modules. The histogram displays the negative log10 of the enrichment *p*-values. The red and black triangles indicate that the corresponding entry has been reported to be associated with colon cancer or other cancer types. The pathways can be divided into five classes according to KEGG Orthology.

CRC is the third most common malignant cancer with the second highest mortality rate (Ayerden et al., 2021; Rydbeck et al., 2021; Peng et al., 2022). By applying CLAM to multi-omics data of CRC (see ‘Data collection and preprocessing’) with KNN ranging from 5 to 15, we obtained 88, 71, 59, 49, 42, 37, 35, 28, 27, 25 and 23 gene modules. TF and KEGG pathway enrichment analyses performed on these modules produced 77 pathways (Supplementary Table S1) and 49 TFs (Supplementary Table S2) shared by different parameters. Representative pathways and TFs are displayed in Figure 3.

According to KEGG Orthology, we divided the pathways into five classes, including proliferation (e.g., cell cycle and DNA replication), immune system (e.g., natural killer cell-mediated cytotoxicity and chemokine signaling pathway), signal transduction (e.g., PI3K/Akt signaling pathway and NF-κB signaling pathway), cancer overview (e.g., proteoglycans in cancer and chemical carcinogenesis) and metabolism (e.g., oxidative phosphorylation and pyruvate metabolism). Additionally, we found that among the 49 TFs, 39 were involved in at least one of the above pathways (see heatmap in Figure 3B). Particularly, 13 TFs were involved in four types of pathways associated with proliferation, immune system, signal transduction and cancer overview, indicating a close correlation between the resulting TFs and pathways.

Following a broad literature exploration, we observed that 67 out of the 77 overlapping pathways were previously reported to be cancer related, in which 55 pathways had been reported in colon cancer and 12 pathways had been reported in other cancer types (Supplementary Table S1). This was reasonable because the overlapping results of different parameters would have been more likely to be studied in previous studies. Additionally, 46 out of the 49 TFs are known to play roles in CRC and the remaining three (RFXAP, SPI1 and RFX5) are related to other cancers (Supplementary Table S2). For example, RELA, NFKB1, NFKBIA and IKBKB are involved in the synthesis and activation of NF-κB, which supports tumorigenesis by promoting cell proliferation, invasion and metastasis and inhibiting apoptosis (Patel et al., 2018). Dysregulation of E2F family (E2F1, E2F3 and E2F4) expression activates or silences oncogenes or tumor suppressors at multiple levels of gene regulation and is involved in CRC progression (Kent and Leone, 2019; Xu et al., 2021). RFXAP, RFX5, RFXANK and CIITA are all associated with MHC II expression, and mutations in any of them lead to MHC II deficiency, which may result in immune evasion in CRC (Michel et al., 2010; Axelrod et al., 2019). Overall, the series of results demonstrated the superior ability of CLAM in reconstructing the modular structure of complex biological systems.

### 3.3 Gene networks associated with CRC survival

**FIGURE 4:**
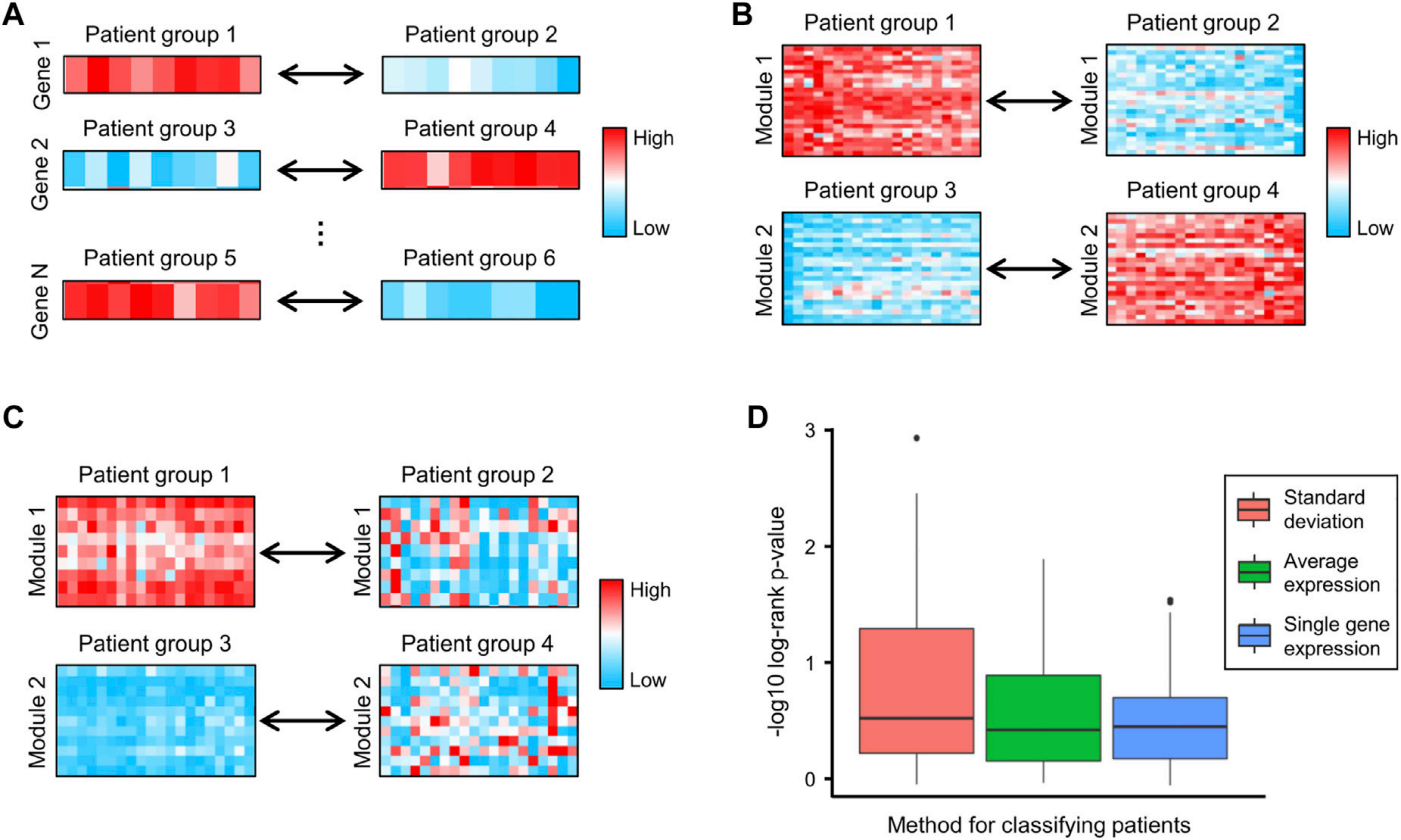
**(A–C)** Examples of classifying patients based on **(A)** the expression of single genes, **(B)** the average expression of genes in each module, and **(C)** the standard deviation of genes in each module, respectively. **(D)** The negative log10 of the log-rank test *p*-values generated by using single gene expression, average expression and standard deviation to classify patients.

**FIGURE 5:**
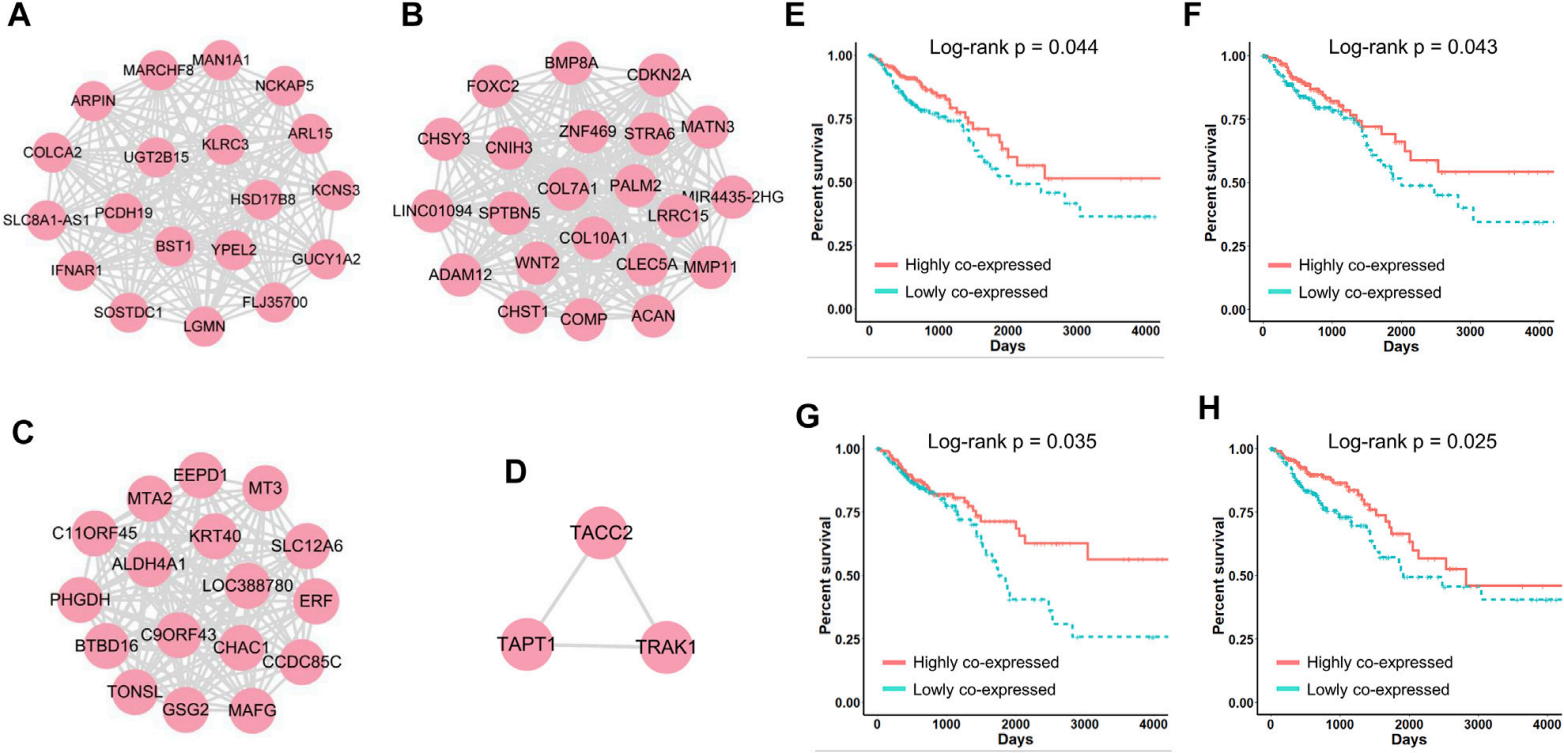
**(A–D)** Four networks correlated with CRC patient survival. **(E–H)** Kaplan‒Meier survival curves generated by performing module-based survival analyses on networks **(A–D)**.

Survival analysis is a cornerstone of medical research, enabling the assessment of clinical outcomes for disease progression and treatment efficiency (Lanczky and Gyorffy, 2021). In traditional survival analysis, patients are divided into low- and high-expression groups based on the expression of a specific gene (Figure 4A) (Figure 5). However, genes are rarely regulated independently and are instead interconnected. A relatively simple way to study the synergistic effects of multiple genes in prognosis is to divide patients based on the average expression of multiple genes. Figure 4B shows two examples of this approach, where genes in Module 1 divide patients into Groups 1 and 2, and genes in Module 2 divide patients into Groups 3 and 4. However, this approach focuses on the differences in gene expression and ignores the correlations between genes. For example, genes in Module 1 are highly expressed in all patients in Group 1 and lowly expressed in all patients in Group 2, suggesting that these genes exhibit significant pairwise expression correlations in both groups of patients. Since many expression correlations arise from functional relationships, the functional relationships in Module 1 are preserved in both Group 1 and Group 2, which limits the difference in survival between Groups 1 and 2. To address this issue, we used the standard deviation of gene expression levels to classify patients. Figure 4C shows two examples of this approach, where genes are highly co-expressed (functionally related) in the left groups and present lower co-expression (dysfunctional) in the right groups.

To compare the above classification criteria, we used the CRC gene modules to classify CRC patients based on individual gene expression, the average expression of genes in each module, and the standard deviation of genes in each module, respectively, and used the log-rank test to assess survival differences. The results showed that classifying patients using the standard deviation yielded the lowest log-rank *p*-values (Figure 4D), indicating that gene co-expression levels were highly correlated with CRC progression and patient survival. We further investigated four survival-related modules (Supplementary Table S3) in which gene–gene co-expression levels were significantly correlated with the overall survival of CRC patients. The network maps and survival curves of these modules are shown in Figure 4. Because co-expressed genes often present functional consistency (Ghazalpour et al., 2006; Kakati et al., 2019), these modules are likely to involve critical gene regulatory or functional relationships affecting the prognosis of CRC.

Evidence has shown that many genes in these modules play crucial roles in cancer progression and can serve as potential prognostic biomarkers. In Figure 4A, HSD17B8 is a good candidate for advanced tumor stages (Luque-García et al., 2010), and COLCA2 is recognized as a colorectal cancer-associated gene (Yin et al., 2022). In Figure 4B, overexpression of COL10A1 enhances the proliferation, migration, and invasion of CRC cells (Huang et al., 2018); MMP11 expression affects the immune response in CRC (Buttacavoli et al., 2021); ADAM12 may play vital roles in the recruitment of immune cells in CRC (Huang et al., 2021); and COMP promotes CRC cell proliferation partially through the activation of the PI3K/Akt/mTOR/p70S6K pathway (Liu et al., 2018). In Figure 4C, ALDH4A1 deficiency leads to the accumulation of proline, which sustains the proliferation and survival of CRC cells (Alaqbi et al., 2022), and MT3 plays a pivotal role in tumor formation, progression, and drug resistance (Si and Lang, 2018). In Figure 4D, TRAK1 is a prognostic biomarker of CRC (An et al., 2011).

Notably, when we performed traditional survival analysis on every individual gene in these networks, only the expression of five genes (CHST1, CHSY3, COMP, MATN3 and PALM2) was significantly correlated with the survival of CRC patients (Supplementary Figure 1). This indicated that in many cases, patient prognosis is not decided by the expression of a single gene but by the synergistic effects of multiple co-regulated genes, which are often neglected by traditional approaches. In summary, with CLAM we defined four networks closely correlated with CRC patient survival, which provided numerous known and novel biomarkers that played critical roles in CRC progression.

## 4 Discussion

With the accumulation of multi-omics expression data, researchers have continuously improved data integration approaches for decades. Nevertheless, most methods were aimed at discovering patient subtypes, and no substantial progress has been made in gene module detection. To address this issue, we developed CLAM, which addressed several critical limitations of data integration and achieved considerable progress in discovering gene regulatory and functional relationships from multi-omics data. However, two issues are worthy of further discussion and research.

First, integration of datasets from different sources is quite time-consuming and requires bulk memory space. Suppose we apply CLAM to three expression matrices $\left(N_{1},M_{1}\right)$ , $\left(N_{2},M_{2}\right)$ and $\left(N_{3},M_{3}\right)$ , where N represent sets of genes, M represents sets of samples, and K represents the KNN parameter. For M≪N and K≪N , the time complexity approximately equals to $O\left({N_{1}}^{2}+{N_{2}}^{2}+{N_{3}}^{2}\right)$ , which is the sum of the time spent by an individual clustering algorithm on multiple datasets. We further compared the running time of CLAM with that of other integrative clustering algorithms. The results showed that CLAM took 15.4 s to perform integrative clustering on the CRC datasets, second only to moCluster (Supplementary Table S4). Considering that moCluster clusters the overlapping genes in the input data (2293 genes), CLAM is the fastest algorithm for clustering the total genes in the input data (12,847 genes). Additionally, algorithm optimization and parallelization can help save computing time. For example, moCluster saves a lot of time by using the consensus PCA algorithm to replace the EM-algorithm used by iCluster (Meng et al., 2016), and parallelization saves nearly half the time for the CLAM algorithm.

Second, methods for integrating gene mutation and expression profiles can be divided into two categories. The first category can predict cancer genes by integrating copy number variations (CNVs) and expression data, such as iPAC (Aure et al., 2013) and NetICS (Dimitrakopoulos et al., 2018). The second category aims to identify cancer driver pathways (or modules) by integrating somatic mutations, CNVs and gene expressions, such as iMCMC (Zhang et al., 2013) and ModulOmics (Silverbush et al., 2019). Different from the integrative clustering algorithms that identify all potential co-expression modules, these methods focus on genes or modules associated with cancer mutations. However, not all cancer genes are associated with cancer mutations. One possible solution is to introduce gene mutation information in the co-expression modules identified by CLAM. In this way, we can identify modules associated with cancer mutations. In subsequent iterations of CLAM, we will explore this algorithm and test its performance.

In addition to improving module detection, this study provides a module-based analysis pipeline to investigate module–disease relationships. With this pipeline we constructed four gene networks significantly correlated with CRC patient survival. Through an extensive literature exploration, we demonstrated that genes in these networks played crucial roles in tumor progression and metastasis. More importantly, we have shown that these results may be missed by traditional survival analysis. With the accumulation of multi-omics data, we believe that module detection and subsequent analysis will attract increasing attention, significantly promoting biomarker discovery in complex diseases.

## Data Availability

The original contributions presented in the study are included in the article/Supplementary Material, further inquiries can be directed to the corresponding author.
