# Supplementary material for: Identification of functional gene modules by integrating multi-omics data and known molecular interactions
Source: Front Genet. 2023 Jan 24;14:1082032. doi: 10.3389/fgene.2023.1082032 (PMC9902936; doi:10.3389/fgene.2023.1082032)
Supplement: Supplementary file 1 [file DataSheet1.PDF]

Supplementary Figures and Tables

Supplementary Table S1 1
Supplementary Table S2 3
Supplementary Table S3 4
Supplementary Table S4 5
Supplementary Figures........ 5

Supplementary Table S1

KEGG pathway enrichment analysis performed on the CRC modules

▲ : Colorectal cancer related △ : Other cancer related ○ : Not reported

CLAM pathway intersection (77)	Cancer_ related	Support
Adrenergic signaling in cardiomyocytes	○	
Aldosterone-regulated sodium reabsorption	○	
Antigen processing and presentation	▲	*https://doi.org/10.1111/imm.12675*
B cell receptor signaling pathway	▲	*https://www.genome.jp/kegg/pathway.html#disease*
Bile secretion	▲	*https://doi.org/10.1016/0959-8049(95)00216-6*
Calcium signaling pathway	▲	*https://www.genome.jp/kegg/pathway.html#disease*
cAMP signaling pathway	▲	*https://www.genome.jp/kegg/pathway.html#disease*
Cell adhesion molecules	▲	*https://www.genome.jp/kegg/pathway.html#disease*
Cell cycle	▲	*https://www.genome.jp/kegg/pathway.html#disease*
cGMP-PKG signaling pathway	▲	*https://pubmed.ncbi.nlm.nih.gov/23804703/*
Chemical carcinogenesis	▲	*https://www.genome.jp/kegg/pathway.html#disease*
Chemokine signaling pathway	▲	*https://www.genome.jp/kegg/pathway.html#disease*
Circadian entrainment	▲	*https://doi.org/10.1158/1541-7786.MCR-10-0086*
Citrate cycle (TCA cycle)	▲	*https://www.genome.jp/kegg/pathway.html#disease*
Cytokine-cytokine receptor interaction	▲	*https://www.genome.jp/kegg/pathway.html#disease*
DNA replication	▲	*http://dx.doi.org/10.1136/gut.42.5.673*
Drug metabolism - cytochrome P450	○	
ECM-receptor interaction	▲	*https://www.genome.jp/kegg/pathway.html#disease*
Endocytosis	▲	*https://doi.org/10.1016/j.ceb.2004.02.003*
Fanconi anemia pathway	▲	*https://pubmed.ncbi.nlm.nih.gov/35330396/*
Fatty acid degradation	▲	*https://www.genome.jp/kegg/pathway.html#disease*
Fc gamma R-mediated phagocytosis	△	*https://pubmed.ncbi.nlm.nih.gov/32676321/*
Focal adhesion	▲	*https://www.genome.jp/kegg/pathway.html#disease*
Gap junction	▲	*https://pubmed.ncbi.nlm.nih.gov/25008544/*
Glycosaminoglycan biosynthesis - chondroitin sulfate / dermatan sulfate	▲	*https://pubmed.ncbi.nlm.nih.gov/22333131/*
Hematopoietic cell lineage	▲	*https://pubmed.ncbi.nlm.nih.gov/25820779/*
Homologous recombination	▲	*https: //doi.org/10.1093/jnci/djab169*
IL-17 signaling pathway	▲	*https://doi.org/10.1016/j.cyto.2018.12.021*
Intestinal immune network for IgA production	▲	*https://pubmed.ncbi.nlm.nih.gov/33707428/*
Leukocyte transendothelial migration	△	*https://pubmed.ncbi.nlm.nih.gov/17163448/*
Metabolism of xenobiotics by cytochrome P450	▲	*https://www.genome.jp/kegg/pathway.html#disease*

Mineral absorption	○	
Mismatch repair	▲	*https://pubmed.ncbi.nlm.nih.gov/20177404/*
mRNA surveillance pathway	△	*https://pubmed.ncbi.nlm.nih.gov/18427545/*
Mucin type O-glycan biosynthesis	▲	*https://pubmed.ncbi.nlm.nih.gov/16741504/*
Natural killer cell mediated cytotoxicity	▲	*https://pubmed.ncbi.nlm.nih.gov/9191519/*
NF-kappa B signaling pathway	▲	*https://www.genome.jp/kegg/pathway.html#disease*
Nicotinate and nicotinamide metabolism	△	*https://pubmed.ncbi.nlm.nih.gov/28515364/*
Nitrogen metabolism	△	*https://pubmed.ncbi.nlm.nih.gov/32302552/*
NOD-like receptor signaling pathway	▲	*https://pubmed.ncbi.nlm.nih.gov/22094258/*
Nucleotide excision repair	▲	*https://doi.org/10.1158/1055-9965.EPI-06-0449*
Olfactory transduction	○	
Osteoclast differentiation	△	*https://pubmed.ncbi.nlm.nih.gov/21514448/*
Ovarian steroidogenesis	○	
Oxidative phosphorylation	▲	*https://www.genome.jp/kegg/pathway.html#disease*
Oxytocin signaling pathway	○	
Pancreatic secretion	○	
Pathways in cancer	▲	*https://www.genome.jp/kegg/pathway.html#disease*
PD-L1 expression and PD-1 checkpoint pathway in cancer	▲	*https://www.genome.jp/kegg/pathway.html#disease*
Peroxisome	▲	*https://pubmed.ncbi.nlm.nih.gov/34996408/*
Phagosome	▲	
PI3K-Akt signaling pathway	▲	*https://www.genome.jp/kegg/pathway.html#disease*
Platelet activation	▲	*https://pubmed.ncbi.nlm.nih.gov/15626589/*
PPAR signaling pathway	▲	*https://www.genome.jp/kegg/pathway.html#disease*
Protein digestion and absorption	○	
Proteoglycans in cancer	▲	*https://www.genome.jp/kegg/pathway.html#disease*
Pyruvate metabolism	△	*https://pubmed.ncbi.nlm.nih.gov/25907297/*
Rap1 signaling pathway	▲	*https://pubmed.ncbi.nlm.nih.gov/28443208/*
Renin secretion	▲	*https://pubmed.ncbi.nlm.nih.gov/20380732/*
Renin-angiotensin system	▲	*https://pubmed.ncbi.nlm.nih.gov/25592047/*
Retinol metabolism	△	*https://pubmed.ncbi.nlm.nih.gov/16530416/*
Ribosome	▲	*https://pubmed.ncbi.nlm.nih.gov/33120992/*
Ribosome biogenesis in eukaryotes	▲	*https://doi.org/10.3390/cells9112361*
RNA degradation	△	*https://pubmed.ncbi.nlm.nih.gov/31724941/*
RNA polymerase	▲	*https://pubmed.ncbi.nlm.nih.gov/21311763/*
Serotonergic synapse	▲	*https://doi.org/10.1016/j.tem.2020.04.008*
Spliceosome	▲	*https://pubmed.ncbi.nlm.nih.gov/34196950/*
Steroid hormone biosynthesis	△	*https://www.genome.jp/kegg/pathway.html#disease*
Sulfur relay system	○	
T cell receptor signaling pathway	▲	*https://www.genome.jp/kegg/pathway.html#disease*
Th1 and Th2 cell differentiation	▲	*https://pubmed.ncbi.nlm.nih.gov/8625361/*
Th17 cell differentiation	▲	*https://pubmed.ncbi.nlm.nih.gov/33495437/*
Thermogenesis	△	*https://pubmed.ncbi.nlm.nih.gov/34071012/*
TNF signaling pathway	▲	*https://pubmed.ncbi.nlm.nih.gov/25561807/*
Valine, leucine and isoleucine degradation	▲	*https://pubmed.ncbi.nlm.nih.gov/32978521/*
Vascular smooth muscle contraction	▲	*https://pubmed.ncbi.nlm.nih.gov/18391202/*
Viral protein interaction with cytokine and cytokine receptor	△	*https://pubmed.ncbi.nlm.nih.gov/22447316/*

Supplementary Table S2

Transcription factor (TF) enrichment analysis performed on the CRC modules

▲ : Colorectal cancer related △ : Other cancer related ○ : Not reported

CLAM TF intersection (49)	Cancer_related	Support
CDX2	▲	*https://pubmed.ncbi.nlm.nih.gov/25663765/*
CEBPA	▲	*https://pubmed.ncbi.nlm.nih.gov/30210916/*
CEBPB	▲	*https://pubmed.ncbi.nlm.nih.gov/33892791/*
CIITA	▲	*https://pubmed.ncbi.nlm.nih.gov/32853948/*
CITED2	▲	*https://pubmed.ncbi.nlm.nih.gov/18054336/*
DEK	▲	*https://pubmed.ncbi.nlm.nih.gov/23902796/*
E2F1	▲	*https://pubmed.ncbi.nlm.nih.gov/26093293/*
E2F3	▲	*https://pubmed.ncbi.nlm.nih.gov/23674142/*
E2F4	▲	*https://pubmed.ncbi.nlm.nih.gov/24100580/*
EGR1	▲	*https://pubmed.ncbi.nlm.nih.gov/24297681/*
EGR2	▲	*https://doi.org/10.1101/2021.02.02.428317*
ETS1	▲	*https://pubmed.ncbi.nlm.nih.gov/12731728/*
ETS2	▲	*https://pubmed.ncbi.nlm.nih.gov/32677671/*
ETV4	▲	*https://pubmed.ncbi.nlm.nih.gov/33648461/*
FOS	▲	*https://pubmed.ncbi.nlm.nih.gov/31338937/*
HIF1A	▲	*https://pubmed.ncbi.nlm.nih.gov/32709922/*
HMGA1	▲	*https://pubmed.ncbi.nlm.nih.gov/22276142/*
HNF4A	▲	*https://doi.org/10.2217/bmm.12.23*
HOXC6	▲	*https://doi.org/10.1158/1538-7445.AM2021-2430*
IKBKB	▲	*https://pubmed.ncbi.nlm.nih.gov/28523736/*
IRF1	▲	*https://pubmed.ncbi.nlm.nih.gov/34313498/*
JUN	▲	*https://pubmed.ncbi.nlm.nih.gov/10874008/*
MYB	▲	*https://pubmed.ncbi.nlm.nih.gov/27622014/*
MYC	▲	*https://pubmed.ncbi.nlm.nih.gov/23237807/*
NFATC1	▲	*https://pubmed.ncbi.nlm.nih.gov/34597678/*
NFATC2	▲	*https://pubmed.ncbi.nlm.nih.gov/30410349/*
NFKB1	▲	*https://pubmed.ncbi.nlm.nih.gov/17852842/*
NFKBIA	▲	*https://pubmed.ncbi.nlm.nih.gov/17354114/*
PAX5	▲	*https://pubmed.ncbi.nlm.nih.gov/32271416/*
PITX2	▲	*https://pubmed.ncbi.nlm.nih.gov/21479692/*
PLAGL1	▲	*https://doi.org/10.3892/ijo.2015.3067*
RELA	▲	*https://pubmed.ncbi.nlm.nih.gov/12827408/*
RFWD2	▲	*https://pubmed.ncbi.nlm.nih.gov/35117395/*
RFX5	△	*https://pubmed.ncbi.nlm.nih.gov/32883983/*
RFXANK	▲	*https://pubmed.ncbi.nlm.nih.gov/11836625/*
RFXAP	△	*https://pubmed.ncbi.nlm.nih.gov/26337469/*
SKIL	▲	*https://pubmed.ncbi.nlm.nih.gov/31471872/*
SP1	▲	*https://pubmed.ncbi.nlm.nih.gov/27434865/*
SPI1	△	*https://pubmed.ncbi.nlm.nih.gov/31774223/*
SRF	▲	*https://pubmed.ncbi.nlm.nih.gov/19082443/*
STAT3	▲	*https://pubmed.ncbi.nlm.nih.gov/16685378/*
STAT5A	▲	*https://pubmed.ncbi.nlm.nih.gov/21826656/*
STAT5B	▲	*https://pubmed.ncbi.nlm.nih.gov/21826656/*

STAT6	▲	https://pubmed.ncbi.nlm.nih.gov/20480530/
TFAP2A	▲	https://pubmed.ncbi.nlm.nih.gov/27916539/
TFAP2B	▲	https://doi.org/10.1158/1541-7786.MCR-20-0867
TP53	▲	https://pubmed.ncbi.nlm.nih.gov/30496442/
YBX1	▲	https://pubmed.ncbi.nlm.nih.gov/28077578/
ZNF148	▲	https://pubmed.ncbi.nlm.nih.gov/28072746/

Supplementary Table S3

Genes in the four survival-related modules

▲: Known prognostic biomarkers ○: Unreported as prognosis-related

Module	Gene	Prognosis-related
A	BST1	○
	COLCA2	▲
	FLJ35700	○
	KCNS3	○
	PCDH19	○
	SOSTDC1	○
	UGT2B15	○
	IFNAR1	○
	GUCY1A2	○
	YPEL2	○
	MAN1A1	○
	LGMN	○
	ARL15	○
	HSD17B8	▲
	KLRC3	○
	MARCHF8	○
	NCKAP5	○
	SLC8A1-AS1	○
	ARPIN	○
B	LINC01094	○
	COL10A1	▲
	CLEC5A	○
	MATN3	○
	MMP11	▲
	LRRC15	○
	MIR4435-2HG	○
	ACAN	○
	CHSY3	○
	COL7A1	○
	BMP8A	○
	CDKN2A	○
	ADAM12	▲
	COMP	▲
	STRA6	○
	PALM2	○

	CNIH3	○
	WNT2	○
	ZNF469	○
	CHST1	○
	SPTBN5	○
	FOXC2	○
C	TACC2	○
	TRAK1	▲
	TAPT1	○
D	ERF	○
	C9ORF43	○
	CHAC1	○
	PHGDH	○
	MT3	▲
	MTA2	○
	TONSL	○
	CCDC85C	○
	GSG2	○
	SLC12A6	○
	ALDH4A1	▲
	EEPD1	○
	MAFG	○
	BTBD16	○
	C11ORF45	○
	KRT40	○
	LOC388780	○

Supplementary Table S4

Running time comparison

Method	Genes under clustering[1]	Time (second)[2]
CLAM (multi-threaded)	12847	15.4
CLAM (single-threaded)	12847	28.6
iNMF	12847	57.3
jNMF	12847	49.5
LemonTree	2293	174.8
moCluster	2293	1.25
iCluster	2293	104.3

[1]Two datasets with a total of 12847 genes and 2293 overlapping genes.

[2]1 core; 11th Gen Intel(R) Core(TM) i5-11300H @ 3.10GHz 16GB RAM.

Supplementary Figures

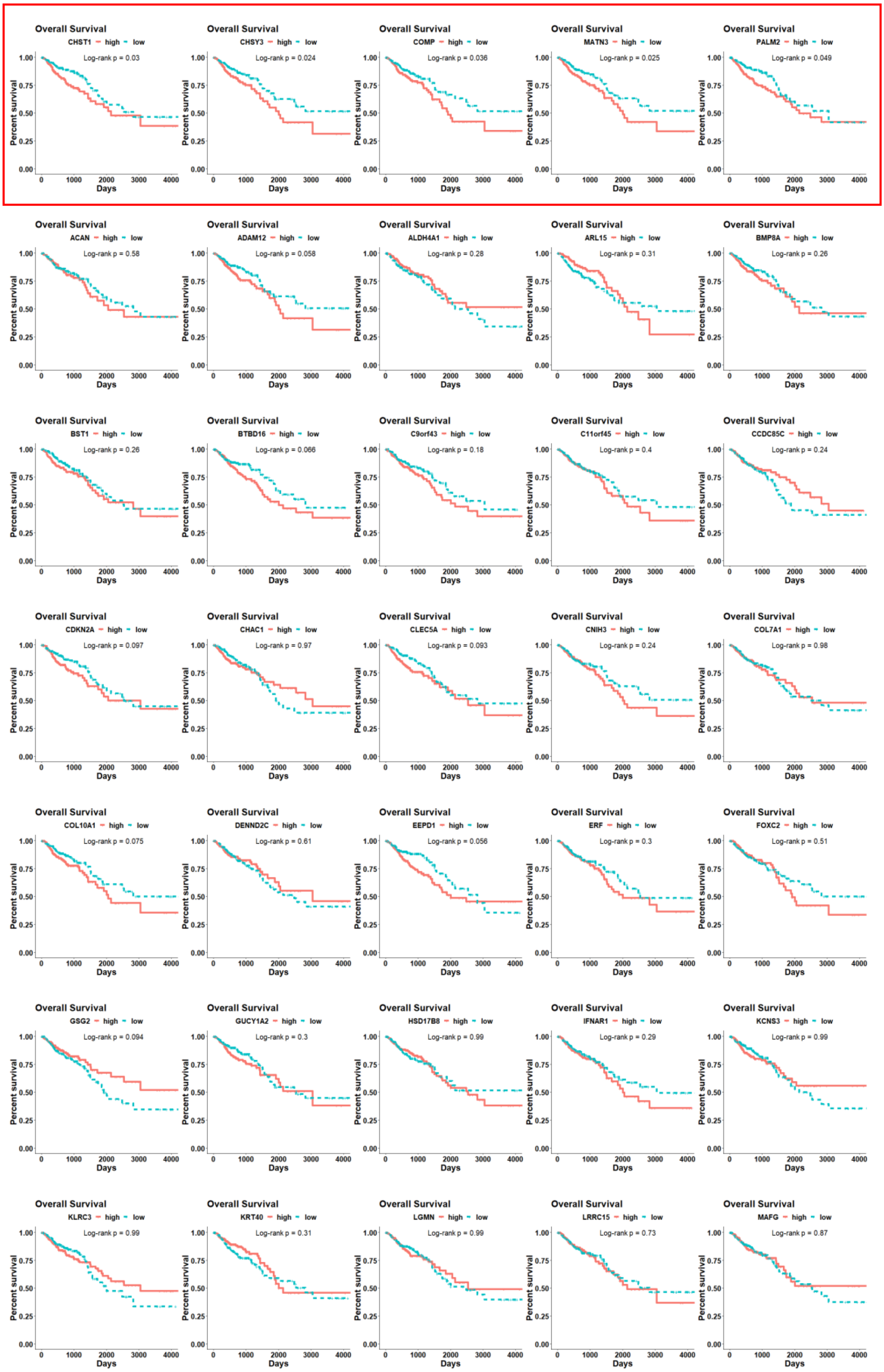
Overall Survival
CHST1 high low
Log-rank p = 0.03
CHSY3 high low
Log-rank p = 0.024
COMP high low
Log-rank p = 0.036
MATN3 high low
Log-rank p = 0.025
PALM2 high low
Log-rank p = 0.049
ACAN high low
Log-rank p = 0.58
ADAM12 high low
Log-rank p = 0.058
ALDH4A1 high low
Log-rank p = 0.28
ARL15 high low
Log-rank p = 0.31
BMP8A high low
Log-rank p = 0.26
BST1 high low
Log-rank p = 0.26
BTBD16 high low
Log-rank p = 0.066
C9orf43 high low
Log-rank p = 0.18
C11orf45 high low
Log-rank p = 0.4
CCDC85C high low
Log-rank p = 0.24
CDKN2A high low
Log-rank p = 0.097
CHAC1 high low
Log-rank p = 0.97
CLEC5A high low
Log-rank p = 0.093
CNIH3 high low
Log-rank p = 0.24
COL7A1 high low
Log-rank p = 0.98
COL10A1 high low
Log-rank p = 0.075
DENND2C high low
Log-rank p = 0.61
EEPD1 high low
Log-rank p = 0.056
ERF high low
Log-rank p = 0.3
FOXC2 high low
Log-rank p = 0.51
GSG2 high low
Log-rank p = 0.094
GUCY1A2 high low
Log-rank p = 0.3
HSD17B8 high low
Log-rank p = 0.99
IFNAR1 high low
Log-rank p = 0.29
KCNS3 high low
Log-rank p = 0.99
KLRC3 high low
Log-rank p = 0.99
KRT40 high low
Log-rank p = 0.31
LGMN high low
Log-rank p = 0.99
LRRC15 high low
Log-rank p = 0.73
MAFG high low
Log-rank p = 0.87
Percent survival
Days

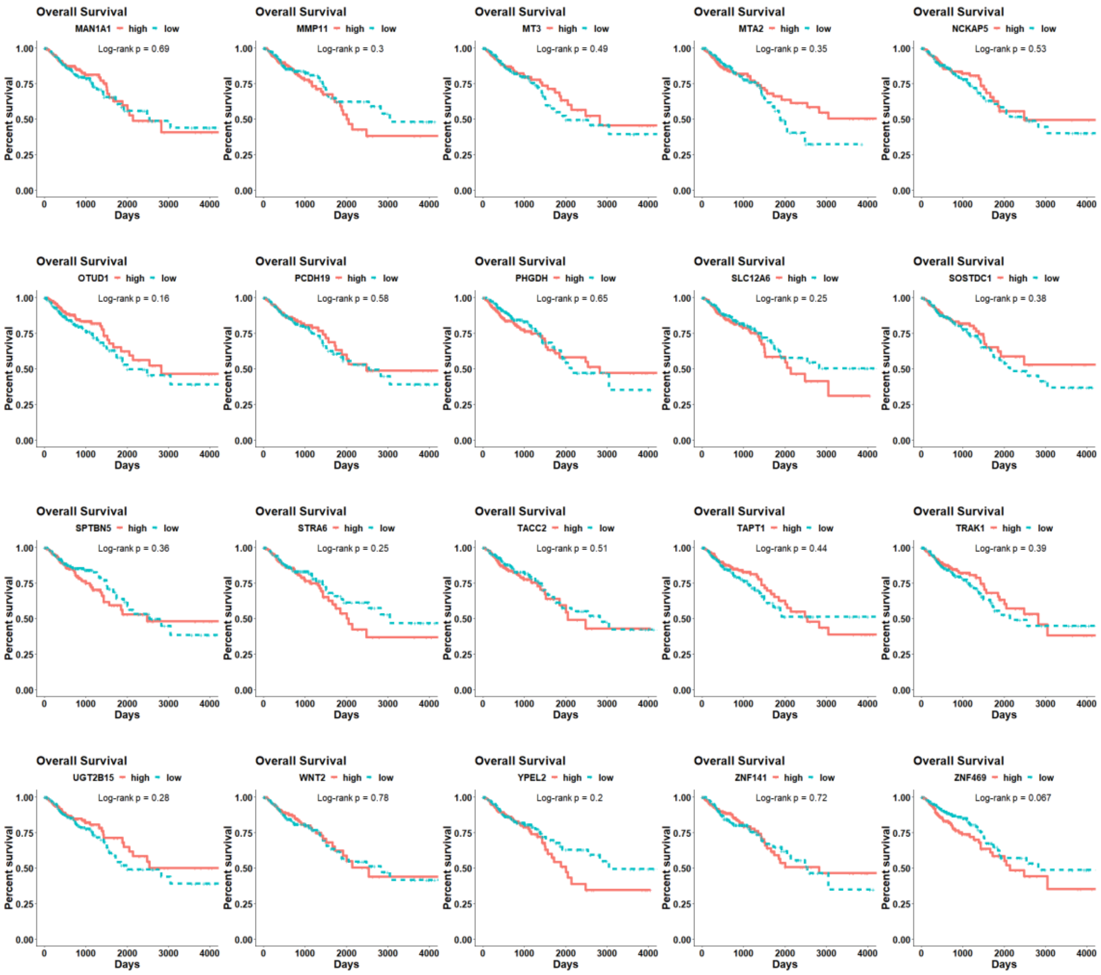

Supplementary Figure 1. Kaplan–Meier survival curves generated by performing survival analysis on each gene in the survival-related networks. The patients were divided according to the expression level of each gene and the overall survival of the two groups were compared by the log-rank test.
